# The persuasiveness of different sources of information on the decision to vaccinate. A cross-sectional study in Germany during the pandemic at the turn of the year 2021/2022

**DOI:** 10.1371/journal.pone.0333268

**Published:** 2025-09-26

**Authors:** Susanne Jordan, Sarah Jane Böttger, Sabine Zinn

**Affiliations:** 1 Robert Koch Institute, Department of Epidemiology and Health Monitoring, Berlin, Germany; 2 Socio-Economic Panel, German Institute for Economic Research, Berlin, Germany; University of Haifa, ISRAEL

## Abstract

Health information about vaccinations is communicated via various sources of information and is crucial for vaccination decisions. Information sources such as interpersonal sources, traditional print and digital media as well as social media offer information about the risks and benefits of vaccination. During health crises such as the COVID-19 pandemic was, some information sources provide hanging or contradictory information, alongside with misinformation and disinformation. Little is known about the relationship between the reported persuasiveness of different sources of information for individual vaccination decisions and differences in this between the vaccinated and unvaccinated. Utilizing data from 10,284 participants in the “Corona-Monitoring Nationwide” survey in Germany from winter 2021/22, this study explored the relationship between the persuasiveness of information sources and vaccination decisions, considering socio-demographic and pandemic-related factors. For more than half of respondents, talks with family, friends, and acquaintances were the most convincing. Traditional media like television and radio were reported by 44%. Newspapers/magazines (online or print) and talks with physicians were each found the most convincing by around one third. About one fifth were persuaded by public authority’s flyers or websites. Less than a tenth each was convinced by social media/messenger services, brochures from pharmacies and health insurances, talks with pharmacy staff or online health portals. Significant differences emerged between vaccinated and unvaccinated individuals. Unvaccinated people were four times more likely to report social media and messenger services as convincing compared to vaccinated people. Reporting talks with doctors and flyers/websites from public authorities as very persuasive significantly reduced the likelihood of being unvaccinated. The findings suggest that in future health crises, information should be disseminated through diverse sources, using both traditional and digital media, as well as interpersonal communication. Proactive science communication on social media and messenger services is crucial to counteract misinformation and disinformation.

## Introduction

Health information and its sources are crucial when people making decisions regarding vaccination [1]. Deciding about vaccination in a health crisis such as the coronavirus pandemic, also known as the COVID-19 pandemic, is a challenge for individuals. At the same time, such societal crises that affect the health of the population pose also a challenge for public health authorities in terms of communicating health information, e. g., communicating risks and benefits [2,3]. During the COVID-19 pandemic, this challenge was particularly true for the preventive measure of vaccinating against severe acute respiratory coronavirus type 2 (SARS-CoV-2) that was a key measure to contain the COVID-19 pandemic [4]. Many people were vaccinated as soon as a vaccine was available, while others hesitated or refused to be vaccinated against COVID-19 [5]. Especially since the COVID-19 pandemic, there has been increased focus on the importance of communication in vaccination decisions, particularly the impact of different sources of information [6–9].

Sources of health information are mass media or interpersonal communication from which health information is obtained. There are a variety of different information sources that are used to provide and disseminate information about immunizations such as COVID-19. In general, studies distinguish between interpersonal sources (physicians, medical and pharmaceutical staff, as well as family, friends and acquaintances), traditional print and digital media (newspaper, television, radio, as well as information from health authorities) and social media (e. g., messenger services, YouTube) [10,11]. Some studies also distinguish between authoritative and non-authoritative sources [12] using different categorizations such as vaccine type, region, and time of study (as older studies do not include Internet sources or social media).

Long-standing research on vaccination has shown that many contextual, personal, and vaccination-specific factors influence individual vaccination decisions, including communication and media [13]. The “three Cs” model of vaccine hesitancy developed by WHO EURO Vaccine Communications Working Group in 2011 summarizes the main influencing factors: 1. trust in the effectiveness and safety of vaccines, in the delivery system and in motivations of policy-makers (confidence); 2. individual risk perception of vaccine-preventable diseases and whether vaccination is considered necessary (complacency); 3. convenience of obtaining the vaccine, i.e., what barriers exist to its availability and affordability (constraints). To achieve broader coverage, the original model has been enhanced by two further factors to the “five Cs” model: 4. the extent of active engagement in information seeking and weighing of cost-benefit (calculation) and 5. the willingness to protect others through one’s own vaccination (collective responsibility) [1]. For the COVID-19 pandemic, more recent research has further refined the model by emphasizing communication by information sources as a separate factor on the vaccination decision [14].

The COVID-19 pandemic was characterized by a constant flow of new or contradictory information regarding the virus’s health implications and transmission, non-pharmaceutical preventative measures, as well as the efficacy and importance of vaccination. This influx of information was paralleled by widespread misinformation and deliberate disinformation campaigns [15]. Social media, in particular, has been suspected of facilitating the spread of misinformation, targeted disinformation, and self-reinforcing groups with conspiracy theories, also known as “echo chambers” [16]. In essence, this situation was described as an “infodemic” amid the pandemic [15,17,18]. The “infodemic” made it challenging for individuals to decide which sources of information about the COVID-19 vaccination were trustworthy. However, trust is crucial for vaccination decisions, especially in decision-making situations of uncertainty, where information is vague or rapidly changing, as was the case during the COVID-19 pandemic [1].

Research on sources of information and their influence on the COVID-19 vaccination decision needs also to consider socio-cultural and regional disparities in information perception and utilization. A study conducted in nine countries across different continents found that television use was associated with a positive COVID-19 vaccination decision in six out of nine countries; the use of print media and official websites was a significant positive predictor in only one and two countries, respectively [19]. Some studies indicate an association between vaccine hesitancy and lower use of websites of public health organizations or institutions [20–22], but frequent use of messaging services [20]. A Japanese study found that interpersonal professional information sources such as physicians and nurses, traditional sources like television as well as digital medical information sites were positively associated with COVID-19 vaccine intention, while interpersonal private sources such as family members, as well as Internet news sites and YouTube increased the chance of intention not to vaccinate [10]. In addition, a few studies indicated that reliance on social media for COVID-19 information correlates with a higher probability of vaccine hesitancy [23–25]. Overall, current research remains inconclusive regarding the influence of information sources on the decision-making process for COVID-19 vaccination.

In Germany, vaccination, along with non-pharmaceutical measures, was a key strategy to contain the COVID-19 pandemic [26]. COVID-19 vaccination has been recommended for the adult population starting in late December 2020 [27] and gradually became available to the whole of the population by autumn 2021 [28]. The European Commission granted a conditional marketing authorization for the COVID-19 vaccine from BioNTech/Pfizer on 21.12.2020. Approvals for other vaccines (e.g., Spikevax/Moderna) followed. By the end of January 2022, about eighty percent of the German population had been vaccinated at least once. This figure results from a combination of estimates derived from register and survey data (76 percent and 92 percent) [29,30]. Ultimately, however at least more than one tenth of the population had been hesitated to get vaccinated by that time.

Common sources of mass information in Germany were government agencies, traditional media such as television, radio, daily newspapers, magazines (online and print) and social media and messenger services. In interpersonal communication, information was disseminated by professionals such as physicians and pharmacists as well as by friends and acquaintances within the private sphere. Early studies on the pandemic indicate that traditional media and their online services were primary sources for information in Germany. Additionally, medical experts and government agencies emerged as key sources of information on COVID-19, vaccination and non-medical protective measures. Social media appeared to play only a subordinate role [7,31,32].

Most studies examined the use or frequency of use of information sources on the COVID-19 vaccination decision. These studies often implicitly assume that the extent of usage reflects the significance of an information source. However, this assumption is flawed because the utilization of an information source does not necessarily signify its perceived importance, nor does considering a source important presuppose its actual usage. For instance, while many people rely on daily news from print or digital media, but it is known that a single or few personal conversations, for example with a physician, can be decisive for a medical decision like vaccination [33]. To tackle this shortcoming of previous studies, we investigate respondents’ self-assessed importance or persuasiveness of an used information source on their COVID-19 vaccination decision. Thus, we gain insights about the actual impact of an information source. So far, still little is known about how convincing information sources are regarding vaccination decisions (and hence their vaccination status), and whether there are differences among different population groups. Concrete, our study aims to examine the following questions: (1) Which sources of information were reported as most persuasive for a COVID-19 vaccination decision in Germany? (2) Do vaccinated and unvaccinated people differ in terms of which sources of information were most persuasive for their vaccination decision?

In our analyses, we consider further factors as covariates that could theoretically influence vaccination decisions following the “five Cs” model outlined above and for which there are findings from other studies that point to an effect [34,35]. These factors include socio-demographics (gender, age, education), health status (which is critical for vaccination eligibility), perceived informedness about the COVID-19 vaccination and concern about the risk of COVID-19 infection.

Our study can contribute to the development of future vaccine communication strategies. Specifically, we can provide recommendations on target group-oriented vaccination communication, especially in time of future pandemics and health crises.

## Materials and methods

### Study design

This study used questionnaire data from the cross-sectional survey of the second wave of the “Corona Monitoring Nationwide” (RKI-SOEP-2) study [36]. It is a cooperative project of the following institutions: The Socio-Economic Panel (SOEP) at the German Institute for Economic Research (DIW Berlin), the Robert Koch Institute (RKI), the Institute for Employment Research (IAB), and the Research Center of the Federal Office for Migration and Refugees (BAMF-FZ). The RKI-SOEP-2 study was conducted within the nationwide population-based SOEP, a longitudinal and multidisciplinary household survey, allowing for representative statements about private households in Germany. The study population of the RKI-SOEP-2 study consisted of individuals aged 14 and older who participated in the 2021 SOEP survey wave. The data collection period spanned from 12 November 2021 to 6 March 2022. For further information see also the detailed study protocol [36].

### Data collection methods

All household members in the SOEP gross sample received an information letter about the study, which also notified them that soon they receive a personal invitation to take part. Subsequently, each target respondent got a small postal package which included the personal invitation and study materials: a study flyer, a letter of motivation from the president of the Robert Koch Institute, a data protection declaration, an informed consent form, a participation plan, and a brief questionnaire. In addition, the package included a kit for SARS-CoV-2 self-testing for laboratory data that were not included in our study. The questionnaire covered topics such as SARS-CoV-2 infection, vaccination against the coronavirus disease, but also, in context of the COVID-19 pandemic, about their health status, health-related behaviours, health information-seeking behaviours, and the impact of the pandemic on daily life. The questionnaire was available in seven languages and could be answered either in written form (PAPI) or online (CAWI). A post-paid monetary incentive (10 euros for adults and 5 euros for adolescents) encouraged participation. All participants received a written notification of the laboratory results of their SARS-CoV-2 antibody self-test results. For further details, see also the study protocol [36].

### Sample characteristics

A total of 11,162 individuals from 6,760 households participated in the RKI-SOEP-2 study. The response rate was 53.7% (according to American Association for Public Opinion Research (AAPOR) 2016 standard [37]). Valid questionnaire data were available from 10,985 subjects (response rate: 51.2%), of these, 10,288 were adults. The four individuals who identified themselves to the category “diverse” could not be included in the analysis due to the small number of cases. Thus, the overall analyses were based on data from a total of 10,284 participants (women: *n* = 5,571, men: *n* = 4,713). This sample size exceeds the minimum required to estimate possible prevalences ranging from 1% to 99% for a target entity to be studied with a confidence level of 95% and a margin of error of 0.1%. The required sample size varies between 380 and 9,600 participants, depending on the assumed prevalence (the related formula can be found in [38]). Thus, the net sample size of the SOEP-RKI-2 study of more than 10,000 participants is sufficient for the planned statistical analysis. Response in the study was not random. For instance, response rates varied by age, with older individuals showing a notably higher participation tendency, and by educational attainment, where individuals with lower education levels participated less frequently than those with higher education. Additional details on response patterns can be found in the study protocol [36]. Survey weights are used in statistical analysis to compensate for the unequal inclusion probabilities in the study sample and the non-random non-response. At this, these survey weights are the product of design weights and non-response adjustment factors; the latter derived through a multistate process by modelling the contactability and the participation of households and its members. To counteract for undercoverage, survey weights were additionally post-stratified to match relevant population distributions provided by the Federal Statistical Office, e.g., age and sex distributions as well as distributions concerning migration figures. For detailed information of the weighting procedure applied along with the variables taken into account for related non-response adjustment as well as for post-stratification, see [39]. The study was approved by the Berlin Chamber of Physicians Ethics Committee (reference ID Eth-33/20 as of September 21, 2021) and all participants provided written informed consent. In the case of minors, both the interviewee and a person with parental authority had to sign the informed consent. The analysis in this paper is limited to persons aged 18 years and older due to different vaccination recommendations between adults and minors in Germany.

### Variables

#### Vaccination status (vaccination decision).

Vaccination status is operationalized by the question: “Have you been vaccinated against the coronavirus disease (COVID-19)?” with the response categories “yes” or “no”. For the purpose of this study, which focuses on vaccination decision, no distinction is made between people who have been vaccinated only once or several times (both one-time and booster vaccinations are included).

#### Survey year participation.

COVID-19 vaccinations have been available throughout the entire field period since the coronavirus vaccines were approved at the end of 2020. To capture the confounding influence of the high prevalence of the more contagious Omicron variant of SARS-CoV-2, which was prevalent in 2022 compared to the Delta variant in 2021, the survey year (i.e., 2021 or 2022) is included as a control variable in the statistical analysis.

#### Persuasiveness of an information source.

The persuasiveness of different information sources for the individual decision to get vaccinated against COVID-19 was surveyed by the question: “What mainly convinced you in your decision for or against COVID-19 vaccination, regardless of your vaccination decision?”. The respondents were given nine different information sources for each of which they were asked to answer this question. These sources were (1) talks with family, friends or acquaintances, (2) television or radio, (3) (online) newspaper or magazines, (4) talks with physicians, (5) flyers or websites from public authorities, (6) social media or messenger services, (7) flyers from pharmacies or health insurances, (8) talks with pharmacists and (9) online health portals. For each information source group five response categories were offered: “not at all true”, “rather not true”, “partially true”, “rather true”, “fully true”. For data analysis, response categories were compiled in the following format: “totally/rather persuasive”, “partly persuasive” and “rather not/not at all persuasive”. This categorization is aimed at enabling a more differentiated analysis, particularly by distinguishing individuals who perceived an information source as only “partly persuasive”—a group that may plausibly differ in their vaccination decision-making from those who found the source clearly persuasive (“fully/rather persuasive”) or not persuasive (“rather not/not at all persuasive”), given their potential ambivalence or mixed feelings toward the information provided.

The covariates in this study included gender, age, education, health status, perceived informedness about the COVID-19 vaccination, and perceived worries about the risk of COVID-19 infection. Since the RKI-SOEP-2 sample is based on the 2021 SOEP cohort, sociodemographic information was at hand and linked to the RKI-SOEP-2 data. Where applicable, our categorizations were based on established standards—for example, ISCED for education and commonly used age groupings. To ensure consistency and interpretability, we applied an uniform coding strategy across all subjective control variables—(self-rated) health status, perceived informedness about COVID-19 vaccination, and perceived worries about the risk of COVID-19 infection—by dichotomizing responses using the top two categories in each case to identify clearly positive or clearly concerned respondents. Although our categorization decisions were based on theoretical considerations and the empirical distribution of responses, we find that they align with the operationalizations used in related studies on COVID-19 vaccine hesitancy.

#### Gender.

Information on gender was surveyed in three categories: female, male, and diverse. For analyses, the latter group was excluded from analysis due to small case size (*n* = 4).

#### Age.

Participant age (in years) was derived from date of birth and the RKI-SOEP-2 survey date. Individuals aged 17 and younger were excluded from analysis (*n* = 697). For analyses, age was categorized into four groups: 18–34 years, 35–49 years, 50–64 years, 65 years and older.

#### Education.

Educational qualification was operationalized according to the International Standard Classification of Education (ISCED) and sorted into three levels: low (ISCED stages 0, 1, 2), medium (ISCED stages 3, 4) and high (ISCED stages 5, 6, 7, 8) [40]. Missing educational data was replaced, if possible, with the most recent available information, e.g., data from the 2020 SOEP wave or earlier. Since the educational level of adults is understood to be a largely stable characteristic, and thus no change was expected due to this characteristic, the Last Observation Carried Forward (LOCF) procedure was used for dealing with missing data. Overall, the number of missing values was reduced from 910 to 395 participants (42%). Because information on educational attainment was still not available for a large number of respondents, a residual category for missing values was formed to accurately represent the sample.

#### Health status.

Self-assessed general health status was collected by asking “How would you describe your current state of health?”. Five response categories (1–5) were possible (five-point Likert scale): “very good”, “good”, fair”, “poor”, “very poor”. The item is widely established, e.g., from World Health Organization (WHO) [41]. For the purpose of our analysis, we created a binary variable by grouping responses into two categories: individuals reporting “very good/good” health were contrasted with those indicating “fair/poor/very poor” health.

#### Perceived informedness.

Perceived informedness about COVID-19 vaccination was captured based on question: “How well or poorly do you feel informed about vaccinating against the coronavirus?”. Participants answered on a five-point Likert-type scale ranging from 1 “very good” to 5 “very poor”. In our analysis, based on the response categories, participants were differentiated in those with “very good/good” versus “fair/poor/very poor” informedness. As a robustness check, we also estimated our models using an alternative specification in which participants were grouped into three categories based on their self-reported level of informedness: “very good/good”, “fair”, and “poor/very poor.”

#### Perceived worries.

People indicated their perceived worries about the risk of a COVID-19 infection by answering the following question: “In the past six months, how often have you worried about whether you or someone close to you might get infected with COVID-19?” The answer categories ranged from 1 “not at all” to 5 “very often”. In our analysis, based on the response categories, participants were differentiated in those who were “very often/often” or “occasionally/rarely/not at all” worried about the risk of COVID-19 infection.

### Statistical analysis

All analyses were conducted using non-response-adjusted and post-stratified survey weights for the RKI-SOEP-2 sample.

Descriptive analyses were performed to examine the frequencies of self-reported persuasiveness of information sources for COVID-19 vaccination, stratified by covariates. Results are presented as weighted percentages with 95% confidence intervals (CI). The assessments of group differences for the persuasiveness of information sources rely on Chi-square tests using survey weights, with statistical significance accepted at *p* *< *0.05. All descriptive analyses were conducted in Stata (version 17).

Overall, the analytic data set comprised 12.1% of data lines (*N* = 1,240) with missing values, primarily due to missing values concerning the information source “talks with pharmacists” (4.1% of all cases) and educational attainment (3.7% of all cases). Little’s test gave strong evidence that the missing data pattern is relevant, i.e., not missing completely at random [42,43]. To counteract problems of selectivity due to item-non-response in regression analysis and to increase statistical power, missing values were imputed. For this multivariate imputation by chained equation (mice) was used [44]. CART (classification and regression trees) constituted the imputation routine for all variables resulting in a total of 30 imputed data sets with 5 iterations in the related Gibbs sampler. To illustrate the structure and patterns of missing data, we generated upset plots and provided them—along with a list of variables used for imputation—on our GitHub repository, see below. Multivariable logistic regression analysis was conducted on the binary outcome variables “not vaccinated” (1) and “vaccinated at least once” (0). The central independent variable was the “persuasiveness of the information source”, which built the basis for examining which of the nine information sources the RKI-SOEP-2 respondents considered relevant for their COVID-19 vaccination decision. In sum, this resulted in nine models to be estimated, for each imputed data set. All models were adjusted for the covariates and year of survey. To ease interpretation all estimated coefficients were transformed to odd ratios (OR). Cluster-robust standard errors accounted for clustering of household members and heteroscedasticity. For regression analysis, the statistical software R (version 4.1.12) was used. In particular, Lumley’s survey package (version 4.1−1) was used for survey weighted logistic regression [45,46] and van Burren’s mice (3.15.0) package for imputation [47] The estimated model results were pooled according to Rubin`s combining rules (7), with own R source code implemented by the authors. Focal variables and covariates were considered significant if the p-value of the related t-test was less than 0.05. All source code for data preparation and statistical analysis as well a supplement on the missing data pattern in the used data set are available at GitHub under https://github.com/bieneSchwarze/vaccinationDecision_infoChannel.

Sensitivity analysis concerning distinct operationalizations of the outcome variable (e.g., the number of vaccine doses received) and the parameterization of the focal outcome variable (e.g., using all five possible values of the scale or three categories) showed no notable differences in the interpretation of effect sizes. All sensitivity analyses are available on request from the authors or can be run via the source code on GitHub.

## Results

### Sample characteristics

The sample of this study consisted of 10,284 adult participants aged 18–99 years (mean age: 50.7 years) who validly completed the survey interview. About 600 people (6%) reported that they had not been vaccinated against COVID-19 at the time of the interview (Table 1).

**Table 1:** Sample description. Source: RKI-SOEP-2, *n *= 10,284.

	Total % (weighted)	Total *n* (unweighted)	Missing % (unweighted)
Gender*			0.0
Men	48.8	4,713	
Women	51.2	5,571	
Age			0.0
18–34 years	24.1	1,887	
35–49 years	22.5	2,420	
50–64 years	27.8	3,450	
65 years and older	25.6	2,527	
Educational level			0.0
Low	9.9	919	
Middle	51.8	4,890	
High	34.6	4,021	
Missing/not assignable	3.7	454	
Health status			0.2
Very good/good	71.5	7,534	
Fair/poor/very poor	28.5	2,731	
Perceived informedness about COVID-19 vaccination			1.0
Very good/good	72.0	7,543	
Fair/poor/very poor	28.0	2,640	
Perceived worries about the risk of COVID-19 infection			0.9
Very often/often	40.2	4,144	
Occasionally/rarely/not at all	59.8	6,045	
COVID-19 vaccination			0.1
Vaccinated at least once	93.95	9,665	
Not vaccinated	6.05	608	
Survey year (participation)			1.6
2021	80.7	7,401	
2022	19.3	2,724	

*Four individuals who identified themselves to the category “diverse” were excluded from analysis due to small number of cases.

### Descriptive results

The results in Table 2 provide insight into the relative persuasiveness of different information sources on individuals’ COVID-19 vaccination decision, regardless of whether the participants decided to vaccinate or not. Among the information sources examined, talks with family, friends or acquaintances were the most frequently reported as the main source of convincing information, with 55.2% of respondents reporting them as such. TV/radio also emerged as a popular source of persuasion, with 44.2% of respondents finding them convincing. About one-third of respondents reported that (online) newspaper or magazines and talks with physicians were the most persuasive sources of information for their vaccination decision, one fifth for flyers/websites from public authorities. In contrast, only about one tenth of the respondents considered information from social media or messenger services or flyers from pharmacies or health insurances to be the most persuasive in their decision-making process regarding the COVID-19 vaccine. Moreover, talks with pharmacists and online health portals were found to be even less influential.

**Table 2:** Persuasiveness of different information sources for the COVID-19 vaccination decision, relative frequency in percent. Source: RKI-SOEP-2, *n* = 10,284.

	Totally/ rather persuasive	Partly persuasive	Rather not/ not at all persuasive
% (95% CI) (weighted)	% (95% CI) (weighted)	% (95% CI) (weighted)
Talks with family/friends/acquaintances	55.2 (53.8–56.7)	22.6 (21.4–23.9)	20.1 (19.9–21.3)
TV/radio	44.2 (42.7–45.7)	25.0 (23.7–26.2)	28.4 (27.1–29.8)
(Online) Newspaper/magazines	35.3 (33.9–36.7)	21.6 (20.5–22.8)	40.3 (38.8–41.7)
Talks with physicians	33.1 (31.8–34.5)	16.3 (15.3–17.5)	47.3 (45.8–48.7)
Flyers/websites from public authorities	20.3 (19.2–21.5)	19.6 (18.5–20.7)	56.8 (55.3–58.2)
Social media/messenger services	9.3 (8.4–10.2)	11.9 (11.0–12.8)	75.3 (74.1–76.5)
Flyers from pharmacies/health insurances	9.0 (8.3–9.9)	13.5 (12.5–14.4)	74.2 (72.9–75.5)
Talks with pharmacists	6.9 (6.2–7.6)	9.1 (8.3–10.0)	79.4 (78.2–80.5)
Online health portals	5.5 (4.8–6.2)	9.9 (9.0–10.8)	80.9 (79.7–82.1)

CI = Confidence interval.

For both vaccinated and unvaccinated individuals, talks with family, friends or acquaintances were the most frequently reported as the main source of persuasive information, with 56.2% and 40.5%, respectively (see Table 3). For vaccinated individuals, TV/radio was the second most convincing source of information with 46.1% reporting it as fully or rather persuasive, for unvaccinated individuals, social media or messenger services were the second most convincing, with 19.5% reporting them as fully or rather persuasive. The unvaccinated were generally less likely than the vaccinated to find the information source persuasive, rejecting most sources as rather not or not persuasive at all, except for social media or messenger services.

**Table 3:** COVID-19 vaccination status by persuasiveness of different information sources for the COVID-19 vaccination decision, relative frequency in percent. Source: RKI-SOEP-2, *n* = 10,284.

	Vaccinated	Unvaccinated	Vaccinated	Unvaccinated	Vaccinated	Unvaccinated
	Fully/rather persuasive		Partly persuasive		Rather not/not at all persuasive	
% (95% CI) (weighted)		% (95% CI) (weighted)		% (95% CI) (weighted)	
Talks with family/friends/acquaintances	56.2 (54.7–57.7)	40.5 (34.6–46.7)	21.9 (20.7–23.2)	33.3 (27.8–39.3)	19.8 (18.6–21.0)	23.6 (18.7–29.3)
TV/radio	46.1 (44.6–47.6)	15.3 (11.9–19.5)	24.1 (22.9–25.4)	38.4 (32.5–44.7)	27.3 (25.9–28.7)	44.6 (38.6–50.7)
(Online) Newspaper/magazines	36.8 (35.3–38.3)	12.9 (9.7–16.8)	21.2 (20.1–22.5)	27.7 (22.6–33.5)	39.1 (37.6–40.7)	56.9 (51.0–62.6)
Talks with physicians	34.4 (33.0–35.9)	12.7 (9.6–16.5)	16.1 (15.0–17.2)	20.9 (16.5–26.2)	46.1 (44.6–47.7)	64.1 (58.5–69.4)
Flyers/websites from public authorities	21.2 (20.0–22.4)	8.0 (5.6–11.2)	19.6 (18.5–20.8)	19.1 (14.9–24.3)	55.9 (54.4–57.4)	69.6 (64.0–74.7)
Social media/messenger services	8.6 (7.8–9.5)	19.5 (15.4–24.3)	10.8 (9.9–11.8)	28.0 (22.7–33.9)	76.9 (75.6–78.1)	50.8 (45.1–56.5)
Flyers from pharmacies/health insurances	9.4 (8.6–10.2)	3.3 (1.8–5.8)	13.5 (12.5–14.5)	13.7 (9.8–18.9)	73.8 (72.4–75.1)	81.2 (75.6–85.8)
Talks with pharmacists	7.2 (6.5–8.0)	2.6 (1.7–4.1)	9.1 (8.3–10.0)	9.6 (6.7–13.6)	79.1 (77.8–80.3)	84.2 (79.8–87.8)
Online health portals	5.6 (4.9–6.4)	4.2 (2.5–6.8)	9.5 (8.6–10.4)	16.3 (12.1–21.5)	81.2 (79.9–82.4)	77.2 (71.5–82.0)

The outcome variable COVID-19 vaccination status is grouped into participants “vaccinated at least once” (*n* = 9,665) or those “not vaccinated” (*n* = 608).

CI = Confidence interval.

### Regression results

Three of the nine information sources surveyed were significantly associated with COVID-19 vaccination status: social media or messenger services, talks with physicians, and information from flyers or websites from public authorities (see Table 4). Unvaccinated individuals were four times more likely to indicate social media or messenger services as totally or rather persuasive (OR=3.8). Those who rated these sources as partly persuasive were still 3.5 times more likely to reject COVID-19 vaccination. Conversely, talks with physicians and information from public authorities’ flyers or websites were significantly more likely to be associated with a positive COVID-19 vaccination status. Unvaccinated individuals were about 0.4-fold and 0.5-fold less likely to consider these sources totally or rather persuasive compared to vaccinated individuals.

**Table 4:** COVID-19 vaccination status by persuasiveness of an information source, sociodemographic and pandemic-related factors among adults in Germany. Source: RKI-SOEP-2, *n* = 10,284.

Information source	Talks with family/friends/ acquaintances	TV/radio	(Online) Newspaper/magazines	Talks with physicians	Flyers/websites from public authorities	Social media/ messenger services	Flyers from pharmacies/health insurances	Talks with pharmacists	Online health portals
	***OR*** (95% CI)	***OR*** (95% CI)	***OR*** (95% CI)	***OR*** (95% CI)	***OR*** (95% CI)	***OR*** (95% CI)	***OR*** (95% CI)	***OR*** (95% CI)	***OR*** (95% CI)
**Persuasiveness of the information source**									
Rather not/not at all persuasive	Ref.	Ref.	Ref.	Ref.	Ref.	Ref.	Ref.	Ref.	Ref.
Partly persuasive	1.30 (0.47–3.60)	1.25 (0.54-2.93)	1.15 (0.53–2.51)	1.04 (0.44–2.45)	0.96 (0.41–2.23)	3.54* (1.36–9.21)	1.35 (0.38–4.81)	1.22 (0.40–3.71)	1.89 (0.77–4.66)
Totally/rather persuasive	0.82 (0.34–1.94)	0.39 (0.15–1.01)	0.45 (0.20–1.04)	0.39** (0.21–0.75)	0.45* (0.20–1.00)	3.80** (1.50-9.64)	0.65 (0.19–2.20)	0.59 (0.29–1.22)	1.07 (0.35– 3.33)
**Gender**									
Male	Ref.	Ref.	Ref.	Ref.	Ref.	Ref.	Ref.	Ref.	Ref.
Female	1.11 (0.51–2.42)	1.10 (0.54–2.26)	1.08 (0.54–2.17)	1.13 (0.55–2.31)	1.13 (0.56–2.30)	1.15 (0.53–2.48)	1.10 (0.53–2.29)	1.10 (0.54–2.22)	1.09 (0.54–2.19)
**Age** (in years)									
18-34	Ref.	Ref.	Ref.	Ref.	Ref.	Ref.	Ref.	Ref.	Ref.
35-49	1.20 (0.39–3.72)	1.23 (0.40–3.79)	1.23 (0.40–3.77)	1.25 (0.41–3.86)	1.20 (0.39–3.71)	1.38 (0.44–4.32)	1.23 (0.40–3.76)	1.23 (0.40–3.86)	1.24 (0.42–3.63)
50-64	0.70 (0.24–2.04)	0.77 (0.26–2.30)	0.73 (0.26–2.09)	0.73 (0.26–2.10)	0.68 (0.23–2.00)	0.82 (0.28–2.43)	0.70 (0.24–1.98)	0.70 (0.24–2.04)	0.70 (0.25–1.94)
65+	0.35 (0.10–1.26)	0.43 (0.12–1.53)	0.39 (0.10–1.44)	0.37 (0.11–1.30)	0.33 (0.09–1.18)	0.43 (0.12–1.59)	0.34 (0.10–1.16)	0.35 (0.10–1.24)	0.34 (0.09–1.22)
**Education**									
Low	1.66 (0.27-10.25)	1.53 (0.26-8.89)	1.53 (0.26-8.83)	1.62 (0.26-10.16)	1.56 (0.25-9.72)	1.26 (0.19-8.53)	1.65 (0.27-10.28)	1.66 (0.26-10.76)	1.64 (0.28-9.69)
Middle	1.34 (0.68–2.64)	1.26 (0.65–2.46)	1.28 (0.65–2.55)	1.38 (0.69–2.76)	1.31 (0.67-2.56)	1.23 (0.59-2.58)	1.34 (0.68–2.66)	1.36 (0.69–2.70)	1.36 (0.70–2.64)
High	Ref.	Ref.	Ref.	Ref.	Ref.	Ref.	Ref.	Ref.	Ref.
**Health status**									
Very good/good	Ref.	Ref.	Ref.	Ref.	Ref.	Ref.	Ref.	Ref.	Ref.
Fair/poor/very poor	0.91 (0.42–1.98)	0.90 (0.43–1.88)	0.91 (0.44–1.88)	0.94 (0.44–1.99)	0.90 (0.43–1.89)	0.97 (0.45–2.10)	0.93 (0.43–1.98)	0.93 (0.44–1.96)	0.94 (0.44–1.99)
**Perceived informedness**									
Very good/good	Ref.	Ref.	Ref.	Ref.	Ref.	Ref.	Ref.	Ref.	Ref.
Fair/poor/very poor	3.03** (1.40–6.59)	2.60* (1.09–6.19)	2.71* (1.19–6.17)	2.86** (1.33–6.13)	2.88** (1.31–6.34)	3.26** (1.43–7.42)	3.10** (1.46–6.57)	3.08** (1.43–6.65)	3.10** (1.42-6.76)
**Perceived worries**									
Occasionally/rarely/not at all	Ref.	Ref.	Ref.	Ref.	Ref.	Ref.	Ref.	Ref.	Ref.
Very often/often	0.32** (0.14–0.71)	0.33** (0.15–0.71)	0.32** (0.15–0.69)	0.34*** (0.16–0.73)	0.33** (0.15–0.70)	0.28** (0.12–0.65)	0.31** (0.14–0.67)	0.31** (0.14–0.68)	0.30*** (0.14–0.65)
**Survey year**									
2021	Ref.	Ref.	Ref.	Ref.	Ref.	Ref.	Ref.	Ref.	Ref.
2022	0.67 (0.34–1.31)	0.64 (0.31–1.33)	0.66 (0.32–1.33)	0.66 (0.33–1.30)	0.66 (0.33–1.30)	0.65 (0.33–1.29)	0.66 (0.33–1.31)	0.66 (0.33–1.31)	0.66 (0.34–1.31)

Outcome variable: negative COVID-19 vaccination status. Nine survey-weighted binary logistic regression models were calculated with one of each of the nine sources of information. Models adjusted for gender, age, education, health status, and survey year. OR = Odds Ratio; CI = Confidence interval; Ref. = Reference category. Bold = *p*-value < 0.05. *** *p < *0.001, ** *p* *< *0.01, * *p* *< *0.05.

Feeling fair, poor, or very poorly informed about COVID-19 vaccination tripled the odds of being unvaccinated across all nine information source groups. In contrast, concerns about contracting COVID-19 reduced the odds by about 0.3-fold. Gender, age, education, health status, and survey timing consistently showed no significant association with COVID-19 vaccination status.

## Discussion

Our study explored the impact of the persuasiveness of information sources on a vaccination decision. At first, we pursued the question: Which sources of information were reported as most persuasive for a COVID-19 vaccination decision in Germany? Our research shows that for the majority of the adult population in Germany, interpersonal sources of information were reported to be the most persuasive in making decisions about COVID-19 vaccination at the turn of the year 2021/2022. Foremost among these sources were talks with family, friends and acquaintances. Additionally, talks with physicians played a pivotal role, reported as most influencing approximately by one-third. Traditional media outlets such as television/radio, (online) newspapers and magazines as well as flyers/websites from public authorities exerted substantial influence as well. In contrast, social media and messenger services played a minor role as information sources for vaccination decisions across the general population.

With our second research question, we examined whether vaccinated and unvaccinated people differ in terms of which sources of information were most persuasive for their vaccination decision. We found relevant differences between vaccinated and unvaccinated individuals in three of the nine types of information sources analysed. For the group of unvaccinated social media and messaging services were reported as highly persuasive sources influencing their decisions compared to the group of vaccinated. In contrast, talks with physicians as well as flyers/websites from public authorities were seen as much less convincing by the unvaccinated group.

The discussion of our results faces the challenge that neither in Germany nor in any other country exactly the same types of information sources on COVID-19 vaccination were analysed. On the one hand, there are a disparity of different research designs and questions hampering unambiguity of findings. Some studies prioritize factors such as trust in health authorities or risk perception [6,48], placing less emphasis on the role of information source. On the other hand, studies focusing on the influence of the source of information on COVID-19 vaccination are challenging to compare due to the varying combinations of sources used in their questionnaires or interviews. In addition, to analyse the relevance of an information source for the vaccination decision, we asked directly how convincing the various sources of information were for the vaccination decision, while in other studies the relevance of information sources was assessed more indirectly. Other studies examined the use or frequency of use (e. g., [7,20,24,32]), the reported level of attention (e. g., [11]) or the reported usefulness (e. g., [6]) regarding different information sources. Furthermore, many studies refer to information on COVID-19 in general (e. g., [49], only a few specifically to the information on the vaccination against COVID-19 (e. g., [7,24,50,51]). Despite the differences in the study surveys, some points should be compared and discussed here. The persuasiveness or the use of information sources is strongly influenced by societal conditions, as demonstrated by the comparative study by Brailovskaia et al. [19]. This observation appears plausible according to the “five C” model for vaccination decisions [1]. Various influencing factors for or against a vaccination decision depend not only on psychological aspects, but also strongly on societal conditions, e.g., trust in the media in general, trust in authorities, the type of risk communication on vaccination or the healthcare system. Accordingly, in this discussion the focus lies as far as possible on studies with populations in Germany.

Our results on the most persuasive information source for a COVID-19 vaccination decision are in line with other results for Germany. The study by Hass et al. also shows private talks to be the most important source of information in Germany [32], while these are of a much lesser relevance in the study by Gehrau et al. [7]. The high importance of traditional media for finding information on COVID-19 vaccination for the general population is also found in other studies with German population groups [7,19,32]. Interpersonal communication with physicians remained important for many in the population during the pandemic [31], but the talks were less frequent compared to both of the sources above. Despite the widespread everyday use of digital services, other studies confirm our finding that social media and messenger services are secondary as a source of information for the general population vaccination [7,31,32].

When comparing the persuasiveness of information sources for vaccination decisions between unvaccinated und vaccinated groups, the picture is different, with significant differences only for three information sources, where social media and messaging services are striking. The digital information source social media and messenger services revealed a high persuasiveness but only for the unvaccinated group, which corresponds with results of the two other smaller studies from Germany [7,32] and other countries [7,20]. However, the cross-country comparison study by Brailovskaia et al. indicates that social media and messenger services are not always or automatically a decisive opinion-former among the unvaccinated or among groups with negative vaccination intentions. They did not find this result for Germany either [19]. Following the “five C model” [1], a central factor appears to be which information sources succeed in conveying trust in the effectiveness and safety of vaccines (confidence), in the distribution system and in credibility of policy-makers. Gehrau et al. were able to demonstrate in their study that trust in alternative sources (such as social media, etc.) was associated with a lower intention to vaccinate against SARS-CoV-2 in Germany [7].

In contrast, talks with physicians as well as flyers/websites from public authorities were seen as much less convincing by the unvaccinated group but persuasive for the vaccinated, who were the large majority of the population at the time of study. It can be assumed that this reflects the unvaccinated groups’ lack of trust in these two professional (interpersonal) sources of information, in opposite to the population majority. Even during the pandemic, physicians and other healthcare professionals remained the most frequent contacts for health information in Germany [31]. Information from health authorities also appeared to be of great importance during the pandemic, which emphasises their role as a trustworthy authority in Germany [31].

Two important findings relating to the confounders analysed should also be emphasised. First, it is notable that the various confounders analysed have no influence on the vaccination decision regarding the differences between vaccinated and unvaccinated people. Second, perceived informedness about COVID-19 vaccination and perceived worries about the risk of COVID-19 infection are important influencing factors for all the sources of information analysed. This finding supports the “five C” model of vaccination behaviour and underscores the importance of risk communication, as individual risk perception is crucial for vaccination decisions (compliance). The fact that unvaccinated people tend to rate themselves as less informed and are less worried about contracting COVID-19 has also been shown in other studies [52], highlighting the need for future information and communication strategies concerning vaccination decision in general.

Overall, the findings highlight the necessity of leveraging a variety of information sources – both traditional and digital – for the general population to overcome vaccine hesitancy in general. It is evident that interpersonal communication should play an important role alongside mass media or social media. Interpersonal communication, such as counselling by physicians, pharmacist or trained peers, offers the potential to convey evidence-based health information in a credible manner during future pandemics and health crises.

Even though the proportion of unvaccinated people in the COVID-19 pandemic remained relatively small, the results on the persuasiveness of social media and messenger services point to an important aspect that is of great importance for future dissemination for vaccination information. In 2022, between 50 and 87% of the population used social media at least occasionally, depending on the study [53,54]. Given the steady rise of social media use [53,54], exposure to information disseminated via these platforms is generally accelerating. This trend is coupled with a surge in misinformation and fake news [55]. The recent pandemic demonstrated that such misinformation often hampers preventive measures like vaccination [56]. Therefore, health authorities need to implement proactive countermeasures during pandemics, which include the rapid dissemination of accurate information through well-structured communication strategies on social media platforms and messaging services. The WHO provides comprehensive guidelines for these efforts [57,58], advocating the use of influencers to promote credible health information and emphasizing the necessity of media literacy training among the public. Furthermore, moderation of content on social media platforms in multiple languages is essential. The WHO also calls on social media companies to commit to removing misinformation, promoting objective health knowledge, and transparently sharing their actions towards these goals. In Germany, public health institutions have already started using social listening for “infodemic” management [59].

Efforts should also focus on bridging knowledge gaps on social media and messaging platforms by providing credible information [51]. It is crucial to tailor the focus and content to the specific target audience of each platform (e.g., young adults at TikTok and older users on Facebook). Information should be age-appropriate, written in simple language, and particularly attentive to vulnerable groups [2]. Tailoring the information dissemination using behavioural and cultural insights is a promising approach recommended by the WHO [60]. Additionally, promoting health literacy regarding vaccination information is another important strategy [61], even though the relationship between health literacy and vaccination has not yet been fully clarified [62].

### Strengths and limitations

Our study distinguishes itself from previous research in two key aspects. First, we employ a representative population-wide study to explore the persuasiveness of information sources on vaccination decisions, accounting for relevant confounding factors such as subjective health status, level of information, and concerns about contracting COVID-19. Second, we move beyond merely examining the use of information sources in decision-making. This approach particularly highlighted the impact of social media and messaging services on negative vaccination decisions. For instance, despite the increasing use of social media and messenger services in Germany, the majority of individuals base their vaccination decisions on information and discussions from private interpersonal communication and traditional print or online information sources. Nonetheless, our findings reveal a strong relevance of social media and messenger services, particularly among the minority who remain unvaccinated. This group, though numerically minor, has significantly reduced the effectiveness of official containment strategies. Notably, our study is the first to identify this effect in a population-representative sample within Germany.

It should also be noted that individual experiences and assessments of the COVID-19 vaccination still have an influence on future vaccination decisions [63]. Therefore, perceived informedness about a specific vaccination and worries about the risk of infection should be considered in future communication strategies.

However, our study has its limitations. We cannot disentangle whether individuals with vaccination hesitancy are inclined to shift away from traditional media towards social media or if social media itself influences vaccination decisions, leading to rejection or delay. To rigorously establish causality in this regard, longitudinal or experimental studies are necessary.

A potential limitation is that participants may not be fully aware of all factors influencing their vaccination decision, which is an inherent challenge when relying on self-reported subjective measures. In addition, there are other possible factors that influence vaccination decisions, but could not be considered in our study, e.g., self-reported frequency of talking or engaging with those sources. Further research should attempt to include these in their analyses. Furthermore, it should be explored whether the various sources of information fulfil different functions for the population in their search for information on vaccination and how this affects their vaccination decision.

Additionally, while the SOEP study design and methodology were carefully developed to minimize biases, some limitations should be acknowledged. The SOEP study employs a diverse sampling strategy to ensure a representative sample of the German population. However, like all longitudinal survey studies, biases may arise from variations in willingness to participate over time and between specific population groups. For example, in the RKI-SOEP sample, response rates were lower among participants with refugee or migration backgrounds and those in full-time employment compared to other groups. Furthermore, a healthy volunteer bias, which leads to an underrepresentation of severely diseased populations, is an inherent limitation of panel studies. These factors could introduce selection bias, particularly among these sub-samples. To address these challenges, regular refreshment samples and high-quality weighting factors were applied to adjust for potential imbalances in the sample. For more details, see the study protocol [36]. Nevertheless, these aspects are not the primary focus of our analysis, as our main objective was to investigate the relationship between information sources and COVID-19 vaccination status. While these potential biases exist, they are unlikely to substantially affect the core findings of our study, as the weighting factors were applied to account for such imbalances and our focus was on population-level trends rather than subgroup-specific conclusions.

## Conclusions

The study investigates how different information sources affected Germans’ decisions regarding the COVID-19 vaccination during the 2020–2022 pandemic, characterised by widespread misinformation. Analysing data from over ten thousand participants in a nationwide representative study in Germany, it reveals that talks with family, friends, and acquaintances wielded the greatest influence, followed by traditional media such as television and radio. Significant differences were observed between vaccinated and unvaccinated individuals, with the latter more likely to be influenced by social media and messenger services and less by talks with physicians as well as flyers/websites from public authorities.

The study underscores the importance of diverse information dissemination strategies across both traditional, digital media but also interpersonal communication sources such as talks with physicians to deliver information and combat misinformation in future vaccination communication strategies. In order to counteract the “infodemic”, i.e., misinformation and disinformation in future pandemics and health-related crises, active science communication measures should be taken in social media and messenger services and a target group-specific approach.

## References

[1] Betsch C, Schmid P, Heinemeier D, Korn L, Holtmann C, Böhm R. Beyond confidence: development of a measure assessing the 5C psychological antecedents of vaccination. PLoS One. 2018;13(12):e0208601. doi: 10.1371/journal.pone.0208601 30532274 PMC6285469

[2] Loss J, Boklage E, Jordan S, Jenny MA, Weishaar H, El Bcheraoui C. Risk communication in the containment of the COVID-19 pandemic: challenges and promising approaches. Bundesgesundheitsblatt Gesundheitsforschung Gesundheitsschutz. 2021;64(3):294–303. doi: 10.1007/s00103-021-03283-3 33564896 PMC7872109

[3] European Centre for Disease Prevention and Control (ECDC). Effective communication around the benefit and risk balance of vaccination in the EU/EEA. Stockholm: ECDC. 2024. https://www.ecdc.europa.eu/en/publications-data/effective-communication-around-benefit-and-risk-balance-vaccination-eueea. Accessed 1 October 2023.

[4] European Centre for Disease Prevention and Control. Factsheet for health professionals on COVID-19.2023. https://www.ecdc.europa.eu/en/infectious-disease-topics/z-disease-list/covid-19/factsheet-covid-19. Accessed 2023 October 1.

[5] Lazarus JV, Wyka K, White TM, Picchio CA, Gostin LO, Larson HJ, et al. A survey of COVID-19 vaccine acceptance across 23 countries in 2022. Nat Med. 2023;29(2):366–75. doi: 10.1038/s41591-022-02185-4 36624316

[6] Muturi N. The Influence of Information Source on COVID-19 Vaccine Efficacy and Motivation for Self-Protective Behavior. J Health Commun. 2022;27(4):241–9. doi: 10.1080/10810730.2022.2096729 35793310

[7] Gehrau V, Fujarski S, Lorenz H, Schieb C, Blöbaum B. The Impact of Health Information Exposure and Source Credibility on COVID-19 Vaccination Intention in Germany. Int J Environ Res Public Health. 2021;18(9):4678. doi: 10.3390/ijerph18094678 33924796 PMC8124400

[8] Nowak GJ, Cacciatore MA. COVID-19 Vaccination and Public Health Communication Strategies: An In-depth Look at How Demographics, Political Ideology, and News/Information Source Preference Matter. Int J Strategic Communication. 2022;16(3):516–38. doi: 10.1080/1553118x.2022.2039666

[9] Allington D, McAndrew S, Moxham-Hall V, Duffy B. Coronavirus conspiracy suspicions, general vaccine attitudes, trust and coronavirus information source as predictors of vaccine hesitancy among UK residents during the COVID-19 pandemic. Psychol Med. 2023;53(1):236–47. doi: 10.1017/S0033291721001434 33843509 PMC8111194

[10] Nomura S, Eguchi A, Yoneoka D, Kawashima T, Tanoue Y, Murakami M, et al. Reasons for being unsure or unwilling regarding intention to take COVID-19 vaccine among Japanese people: A large cross-sectional national survey. Lancet Reg Health West Pac. 2021;14:100223. doi: 10.1016/j.lanwpc.2021.100223 34368797 PMC8324415

[11] Yang X, Wei L, Liu Z. Promoting COVID-19 Vaccination Using the Health Belief Model: Does Information Acquisition from Divergent Sources Make a Difference?. Int J Environ Res Public Health. 2022;19(7):3887. doi: 10.3390/ijerph19073887 35409568 PMC8997454

[12] Yoneoka D, Eguchi A, Nomura S, Kawashima T, Tanoue Y, Murakami M, et al. Identification of optimum combinations of media channels for approaching COVID-19 vaccine unsure and unwilling groups in Japan. Lancet Reg Health West Pac. 2022;18:100330. doi: 10.1016/j.lanwpc.2021.100330 34927110 PMC8665235

[13] MacDonald NE, SAGE Working Group on Vaccine Hesitancy. Vaccine hesitancy: Definition, scope and determinants. Vaccine. 2015;33(34):4161–4. doi: 10.1016/j.vaccine.2015.04.036 25896383

[14] Razai MS, Oakeshott P, Esmail A, Wiysonge CS, Viswanath K, Mills MC. COVID-19 vaccine hesitancy: the five Cs to tackle behavioural and sociodemographic factors. J R Soc Med. 2021;114(6):295–8. doi: 10.1177/01410768211018951 34077688 PMC8209756

[15] Wilhelm E, Ballalai I, Belanger M-E, Benjamin P, Bertrand-Ferrandis C, Bezbaruah S, et al. Measuring the Burden of Infodemics: Summary of the Methods and Results of the Fifth WHO Infodemic Management Conference. JMIR Infodemiology. 2023;3:e44207. doi: 10.2196/44207 37012998 PMC9989916

[16] Jennings W, Stoker G, Bunting H, Valgarðsson VO, Gaskell J, Devine D, et al. Lack of Trust, Conspiracy Beliefs, and Social Media Use Predict COVID-19 Vaccine Hesitancy. Vaccines (Basel). 2021;9(6):593. doi: 10.3390/vaccines9060593 34204971 PMC8226842

[17] Briand SC, Cinelli M, Nguyen T, Lewis R, Prybylski D, Valensise CM, et al. Infodemics: A new challenge for public health. Cell. 2021;184(25):6010–4. doi: 10.1016/j.cell.2021.10.031 34890548 PMC8656270

[18] World Health Organization (WHO). Infodemic. No date. Geneva: https://www.who.int/health-topics/infodemic#tab=tab_1 Accessed 2023 October 16.

[19] Brailovskaia J, Schneider S, Margraf J. To vaccinate or not to vaccinate!? Predictors of willingness to receive Covid-19 vaccination in Europe, the U.S., and China. PLoS One. 2021;16(12):e0260230. doi: 10.1371/journal.pone.0260230 34851986 PMC8635370

[20] de Vries H, Verputten W, Preissner C, Kok G. COVID-19 Vaccine Hesitancy: The Role of Information Sources and Beliefs in Dutch Adults. Int J Environ Res Public Health. 2022;19(6):3205. doi: 10.3390/ijerph19063205 35328892 PMC8948729

[21] Reno C, Maietti E, Di Valerio Z, Montalti M, Fantini MP, Gori D. Vaccine Hesitancy towards COVID-19 Vaccination: Investigating the Role of Information Sources through a Mediation Analysis. Infect Dis Rep. 2021;13(3):712–23. doi: 10.3390/idr13030066 34449654 PMC8395997

[22] Park S, Massey PM, Stimpson JP. Primary Source of Information About COVID-19 as a Determinant of Perception of COVID-19 Severity and Vaccine Uptake: Source of Information and COVID-19. J Gen Intern Med. 2021;36(10):3088–95. doi: 10.1007/s11606-021-07080-1 34378115 PMC8354304

[23] Kim J, Kim Y, Li Y. Source of information on COVID-19 vaccine and vaccine hesitancy among U.S. Medicare beneficiaries. J Am Geriatr Soc. 2022;70(3):677–80. doi: 10.1111/jgs.17619 34894409

[24] Osuagwu UL, Mashige KP, Ovenseri-Ogbomo G, Envuladu EA, Abu EK, Miner CA, et al. The impact of information sources on COVID-19 vaccine hesitancy and resistance in sub-Saharan Africa. BMC Public Health. 2023;23(1):38. doi: 10.1186/s12889-022-14972-2 36609264 PMC9816548

[25] Sieber WJ, Achar S, Achar J, Dhamija A, Tai-Seale M, Strong D. COVID-19 vaccine hesitancy: Associations with gender, race, and source of health information. Fam Syst Health. 2022;40(2):252–61. doi: 10.1037/fsh0000693 35446060

[26] Grote U, Arvand M, Brinkwirth S, Brunke M, Buchholz U, Eckmanns T, et al. Measures to cope with the COVID-19 pandemic in Germany: nonpharmaceutical and pharmaceutical interventions. Bundesgesundheitsblatt Gesundheitsforschung Gesundheitsschutz. 2021;64(4):435–45. doi: 10.1007/s00103-021-03306-z 33787944 PMC8010780

[27] Vygen-Bonnet S, Koch J, Bogdan C, Harder T, Heininger U, Kling K. Beschluss der STIKO zur 1. Aktualisierung der COVID-19-Impfempfehlung und die dazugehörige wissenschaftliche Begründung. Epid Bull. 2021(2):64–132. doi: 10.25646/7820

[28] Waize M, Scholz S, Wichmann O, Harder T, Treskova-Schwarzbach M, Falman A et al. Die Impfung gegen COVID-19 in Deutschland zeigt eine hohe Wirksamkeit gegen SARS-CoV-2-Infektionen, Krankheitslast und Sterbefälle. Epid Bull. 2021;35:3–10. doi: 10.25646/8887

[29] Robert Koch Institut (RKI). COVID-19-Impfungen in Deutschland. Berlin: Zenodo. 2024.

[30] Robert Koch Institut (RKI). COVID-19 Impfquoten-Monitoring in Deutschland (COVIMO) – 10. Report (Datenerhebung: 10.01.22 - 27.01.22). 2022. https://www.rki.de/DE/Content/InfAZ/N/Neuartiges_Coronavirus/Projekte_RKI/COVIMO_Reports/covimo_studie_bericht_8.html?nn=2444038

[31] Link E, Czerwinski F, Calhoun K, Grimm M, Großmann U, Baumann E. Hat sich das Informationsverhalten zu Gesundheitsthemen in der Corona-Pandemie verändert? Teilergebnisse der Studie “HINTS Germany. Trendmonitor. 2022(6):1–5.

[32] Haß W, Orth B, von Rüden U. COVID-19 vaccination status, sources of used information and socio-demographic characteristics-results of the CoSiD study. Bundesgesundheitsblatt Gesundheitsforschung Gesundheitsschutz. 2023;66(8):846–56. doi: 10.1007/s00103-023-03736-x 37438645 PMC10371914

[33] Frank E, Dresner Y, Shani M, Vinker S. The association between physicians’ and patients’ preventive health practices. CMAJ. 2013;185(8):649–53. doi: 10.1503/cmaj.121028 23569163 PMC3652935

[34] Betsch C, Eitze S, Felgendreff L, Geiger M, Korn L, Schmid P, et al. Impfungen. COSMO. COVID-19 Snapshot Monitoring. https://projekte.uni-erfurt.de/cosmo2020/web/topic/impfung/10-impfungen/ 2022. Accessed 2024 May 1.

[35] Allington D, Duffy B, Wessely S, Dhavan N, Rubin J. Health-protective behaviour, social media usage and conspiracy belief during the COVID-19 public health emergency. Psychol Med. 2021;51(10):1763–9. doi: 10.1017/S003329172000224X 32513320 PMC7298098

[36] Bartig S, Brücker H, Butschalowsky H, Danne C, Gößwald A, Goßner L, et al. Corona Monitoring Nationwide (RKI-SOEP-2): Seroepidemiological Study on the Spread of SARS-CoV-2 Across Germany. J Economics Statistics [Jahrbücher für Nationalökonomie und Statistik]. 2023;3-4(431–449). doi: 10.1515/jbnst-2022-0047

[37] American Association for Public Opinion Research AAPOR. Standard definitions. Final dispositions of case codes and outcome rates for surveys. Revised 2023. 2023. https://aapor.org/wp-content/uploads/2024/03/Standards-Definitions-10th-edition.pdf. Accessed 2025 September 1. https://www.aapor.org/AAPOR_Main/media/publications/Standard-Definitions20169theditionfinal.pdf

[38] Cochran WG. Sampling Techniques. 3rd Edition ed. New York: John Wiley & Sons. 1977.

[39] Danne C, Priem M, Steinhauer HW. SOEP-Core – 2021: Sampling, nonresponse, and weighting in wave 2 of living in Germany – Nationwide Corona-Monitoring (RKI-SOEP2). SOEP Survey Papers. Series C. 2022.

[40] Statistics UIf. International Standard Classification of Education. ISCED 2011. UNESCO Institute for Statistics. 2012.

[41] De Bruin A, Picavet H, Nossikov A. Health interview survey. Towards harmonization of methods and instruments. Copenhagen: WHO Regional Office for Europe. 1996. 8857196

[42] Little RJ, Rubin DB. Statistical analysis with missing data. John Wiley & Sons. 2019.

[43] Little RJ. A test of missing completely at random for multivariate data with missing values. J Am Stat Assoc. 1988;83(404):1198–202.

[44] van Buuren S, Groothuis-Oudshoorn K. Mice: multivariate imputation by chained equations in R. J Stat Soft. 2011.

[45] Lumley T. Analysis of complex survey samples. J Stat Soft. 2004;9(8). doi: 10.18637/jss.v009.i08

[46] Lumley T. R package survey: Analysis of complex survey samples. 2021. http://r-survey.r-forge.r-project.org/. Accessed 2025 September 1.

[47] Van Buuren S, Groothuis-Oudshoorn K, Vink G, Schouten R, Robitzsch A, Rockenschaub P, et al. R package mice: Multivariate imputation by chained equations. Version 3.15.0. https://cran.r-project.org/web/packages/mice/index.html 2022. Accessed 2025 September 1.

[48] El-Far Cardo A, Kraus T, Kaifie A. Factors that shape people’s attitudes towards the COVID-19 pandemic in Germany–The influence of Media, politics and personal characteristics. Int J Environ Res Public Health. 2021;18(15):7772. doi: 10.3390/ijerph18157772 34360063 PMC8345618

[49] Meppelink CS, Bos L, Boukes M, Möller J. A health crisis in the age of misinformation: how social media and mass media influenced misperceptions about COVID-19 and compliance behavior. J Health Commun. 2022;27(10):764–75. doi: 10.1080/10810730.2022.2153288 36576116

[50] Yoda T, Suksatit B, Tokuda M, Katsuyama H. The relationship between Sources of COVID-19 vaccine information and willingness to be vaccinated: an internet-based cross-sectional study in Japan. Vaccines (Basel). 2022;10(7):1041. doi: 10.3390/vaccines10071041 35891205 PMC9320181

[51] Piltch-Loeb R, James R, Albrecht SS, Buttenheim AM, Dowd JB, Kumar A, et al. What Were the Information Voids? A Qualitative Analysis of Questions Asked by Dear Pandemic Readers between August 2020-August 2021. J Health Commun. 2023;28(sup1):25–33. doi: 10.1080/10810730.2023.2214986 37390014

[52] Santirocchi A, Spataro P, Costanzi M, Doricchi F, Rossi-Arnaud C, Cestari V. Predictors of the Intention to Be Vaccinated against COVID-19 in a Sample of Italian Respondents at the Start of the Immunization Campaign. J Pers Med. 2022;12(1):111. doi: 10.3390/jpm12010111 35055426 PMC8780740

[53] Koch W. Soziale Medien werden 30 Minuten am Tag genutzt – Instagram ist die Plattform Nummer eins. Ergebnisse der ARD/ZDF-Onlinestudie 2023. [Social media is used for 30 minutes a day – Instagram is the number one platform Results of the ARD/ZDF online study 2023]. Media Perspektiven. 2023; 26(November):1-–8. Available from: https://www.ard-zdf-onlinestudie.de/

[54] We Are Social, Meltwater. Digital 2022: Germany 2022. https://datareportal.com/reports/digital-2022-germany. Accessed 1 September 2025.

[55] Aïmeur E, Amri S, Brassard G. Fake news, disinformation and misinformation in social media: a review. Soc Netw Anal Min. 2023;13(1):30. doi: 10.1007/s13278-023-01028-5 36789378 PMC9910783

[56] Recio-Román A, Recio-Menéndez M, Román-González MV. Influence of media information sources on vaccine uptake: The full and inconsistent mediating role of vaccine hesitancy. Computation. 2023;11(10):208.

[57] Rubinelli S, Purnat TD, Wilhelm E, Traicoff D, Namageyo-Funa A, Thomson A, et al. WHO competency framework for health authorities and institutions to manage infodemics: its development and features. Hum Resour Health. 2022;20(1):35. doi: 10.1186/s12960-022-00733-0 35525924 PMC9077350

[58] World Health Organization (WHO), United Nations Children’s Fund (UNICEF). How to build an infodemic insights report in six steps. Geneva: WHO and UNICEF. 2023. https://www.who.int/publications/i/item/9789240075658. Accessed 12 September 2025.

[59] Boender TS, Schneider PH, Houareau C, Wehrli S, Purnat TD, Ishizumi A, et al. Establishing Infodemic Management in Germany: A Framework for Social Listening and Integrated Analysis to Report Infodemic Insights at the National Public Health Institute. JMIR Infodemiology. 2023;3:e43646. doi: 10.2196/43646 37261891 PMC10273031

[60] Thomson A, Vallée-Tourangeau G, Suggs LS. Strategies to increase vaccine acceptance and uptake: From behavioral insights to context-specific, culturally-appropriate, evidence-based communications and interventions. Vaccine. 2018;36(44):6457–8. doi: 10.1016/j.vaccine.2018.08.031 30201305

[61] Biasio LR. Vaccine hesitancy and health literacy. Hum Vaccin Immunother. 2017;13(3):701–2. doi: 10.1080/21645515.2016.1243633 27808587 PMC5360145

[62] Lorini C, Santomauro F, Donzellini M, Capecchi L, Bechini A, Boccalini S, et al. Health literacy and vaccination: A systematic review. Hum Vaccin Immunother. 2018;14(2):478–88. doi: 10.1080/21645515.2017.1392423 29048987 PMC5806657

[63] Sprengholz P, Henkel L, Böhm R, Betsch C. Historical narratives about the COVID-19 pandemic are motivationally biased. Nature. 2023;623(7987):588–93. doi: 10.1038/s41586-023-06674-5 37914928

